# The Mediating Role of Worker-Occupation Fit in the Relationship Between Occupational Stress and Depression Symptoms in 1988 Medical Workers: A Cross-Sectional Study

**DOI:** 10.3389/fpubh.2022.843845

**Published:** 2022-05-17

**Authors:** Ruican Sun, Keyao Lv, Zirui He, Liang Liao, Hongping Wang, Yajia Lan

**Affiliations:** ^1^Department of Preventive Medicine, School of Public Health, Chengdu Medical College, Chengdu, China; ^2^Department of Environmental Health and Occupational Medicine, West China School of Public Health and West China Fourth Hospital, Sichuan University, Chengdu, China

**Keywords:** depression symptoms, occupational stress, worker occupation fit, medical workers, mediate effect

## Abstract

**Objective:**

Occupational stress is generally acknowledged as a global phenomenon with significant health and economic consequences. The medical worker is a vulnerable group at a high-level risk for depression symptoms. This study aimed to examine the mediating effect of worker-occupation fit (WOF) in relation to occupational stress and depression symptoms among 1988 medical workers in China.

**Methods:**

A multi-center cross-sectional study was conducted during June and October 2020 in Henan Province, China. The participants were medical workers from four targeted hospitals (included one general and three specialized hospitals). The Depression, Anxiety, and Stress Scale (DASS-21 Scale), Worker-Occupation Fit Inventory (WOFI), as well as questions about demographic and occupational information were administered in questionnaires distributed to 1988 medical workers. Hierarchical linear regression analysis was used to examine the mediating role of worker occupation fit.

**Results:**

In this study, there are 43.5% (*n* = 864) of medical workers experienced depression symptoms. The mean score of WOF was 31.6 ± 7.1, characteristic fit, need supply fit and demand ability fit were 11.3 ± 2.5, 10.1 ± 2.7, 12.9 ± 2.2, respectively. The occupational stress was negatively related to worker occupation fit (*r* = −0.395, *P* < 0.001), characteristic fit (*r* = −0.529, *P* < 0.001), need supply fit (*r* = −0.500, *P* < 0.001), and demand ability fit (*r* = −0.345, *P* < 0.001). The occupational stress and depression symptoms have a positive relationship (*r* = 0.798, *P* < 0.001). The proportion of worker occupation fit mediation was 6.5% of total effect for depression symptoms.

**Conclusion:**

Occupational stress has been identified as a risk factor for depression symptoms. Practical strategies for improving medical workers' WOF level would help them better cope with various work-related stressors to reduce depression symptoms. Hospital administrators could reduce medical workers' depression symptoms by taking comprehensive measures to improve the WOF.

## Introduction

Occupational stress involves all workplaces, which is generally acknowledged as a global phenomenon with significant health and economic consequences in both developed and developing countries (1). The current work style and intensity of society will continue to increase the occurrence and development of occupational stress, and may even induce a variety of stress-related diseases. Occupational stress is associated with increased risk of mental disorders, [i.e., depression (2), burnout (3)], somatic diseases [i.e., insomnia (4), hypertension (5)], and organizational outcomes [i.e., job satisfaction (6), wellness (7)]. In recent decades, occupational stress represents a large, complex and costly phenomenon in the workplace worldwide (8).

Medical workers is widely regarded as one of the most challenging professions. Medical workers often receive strict and long-term training to achieve their professional goals (9). Therefore, medical workers is a vulnerable group at a high-level risk for mental illnesses (10). Studies reported that a total of forty-six nurses suicide induced by anxiety/depression in China from 2007 to 2016 (11), as were a total of fifty-one physicians suicide induced by anxiety/depression from 2008 to 2016 (12). The National Institute for Occupational Safety and Health suggested that medical workers have the common stressors in hospital settings consist of inadequate staffing levels, long work hours, shift work, role ambiguity, and exposure to infectious diseases (13). Another national research reported that higher staff turnover, more absences due to illness, decreased performance, and more complaints and grievances were signs of stress (14). The above-mentioned reports, medical workers have a higher risk of mental disorders, and many countries have begun to pay attention to the prevention of mental disorders in the occupational environment.

Depression is a major mental health issue in the global world, which has long been a topic attracting much interest across a wide range of research fields. The relationship between occupational stress and depression symptoms is also an ongoing research issue. There are plenty of studies in various populations suggested that psychological capital (15, 16), social support (17), coping style (18), work demand (19) and work-family conflict (20) are considered as the mediating role on occupational stress and mental disorders. Previous reports highlighted that occupational stress affects depression symptoms, and these vulnerable individuals need more support. Li et al. examined psychological capital significantly mediated the associations of occupational stress and depression symptoms among Chinese physicians (21). Prerna Varma et al. found that poor sleep quality, lower levels of resilience, younger age and loneliness significantly mediated the links between stress and depression among young adults (22). Wang et al. (23) explored the mediating role of resilience between occupational stress and depression in female nurses, and the results suggested that mental elasticity has an indirect effect on occupational stress and depression. The mediating effect of mental elasticity accounted for 16.08% of the total effect. Batalla et al. (24) examined the correlation between spirituality and depression and found the moderating effect of occupational stress in registered nurses. Taken together, there are not only direct effects but also mediating effects between occupational stress and depression symptoms.

Worker-occupation fit (WOF) was defined as the fit level of the worker's characteristic, need, and ability with the occupational environment's culture, supply, and demand (25). In our early research, we constructed a theoretical model of the WOF effects on occupational stress and related disorders (26). According to the definition of WOF, it was divided into three types, namely, characteristic fit (CF), need supply fit (NSF) and demand ability fit (DAF). WOF was derived from the theory of personal environment fit. Since the 1980s, the study of person-environment fit has begun to emerge as a research hot-spot in organizational behaviors and organizational management, with most studies focusing on the relationship between organizational outcomes and personal environment fit (27–29). Many empirical studies were conducted in this area to find a better fit level to explore the positive effects on job attitudes (30), organizational citizenship behaviors (31), and job performance (32). The better fit level between individual and occupational environment, which can improve the higher levels of organizational citizenship behavior, led to fewer work errors and more harmonious interpersonal relationships. Therefore, the fit of the individual (i.e., needs, values, abilities, and personal characteristics) with the occupational environment (i.e., values, rewards, job requirements, culture, and physical environment) is an important factor influencing the worker's attitudes and behaviors in their workplaces (33). At present, there has few studies focuses on the association of WOF and mental disorders for medical workers.

Furthermore, WOF is most consistent with the theoretical implications of occupational stress, which is measured as, a negative physical, psychological, and behavioral response that occurs when a worker's resources, skills, and needs do not match the job demands (34, 35). Our preliminary findings showed a significant negative association between WOF and occupational stress, that is, higher levels of WOF lead to lower levels of occupational stress (25). Based on our preliminary study, it would be valuable to further explore the effect of WOF on depression symptoms and to further explore whether there is a mediating role between them.

To sum up, whether there is a correlation between WOF and depression symptoms, and whether WOF plays a mediating role between occupational stress and depression symptoms that it worth to confirm. In short, this study aimed to examine the mediating effect of WOF on occupational stress and depression symptoms in 1988 medical workers to provide scientific evidence for developing effective interventions to improve depression symptoms.

## Methods

### Participants and Procedures

A cross-sectional study was conducted in Henan Province by multi-center survey from June to October, 2020. The participants were medical workers from four targeted hospitals (included a comprehensive grade 3A hospital, a psychiatric hospital, a children's hospital and an infectious hospital). Based on the study design, the sample size calculated by Fisher's method (36). The sample size was estimated to be 502 participants. Considering there are many research variables in this study, a design coefficient of 1.3 was added into the final sample size. Therefore, the final sample size calculated was 653 participants.

**Figure 1:**
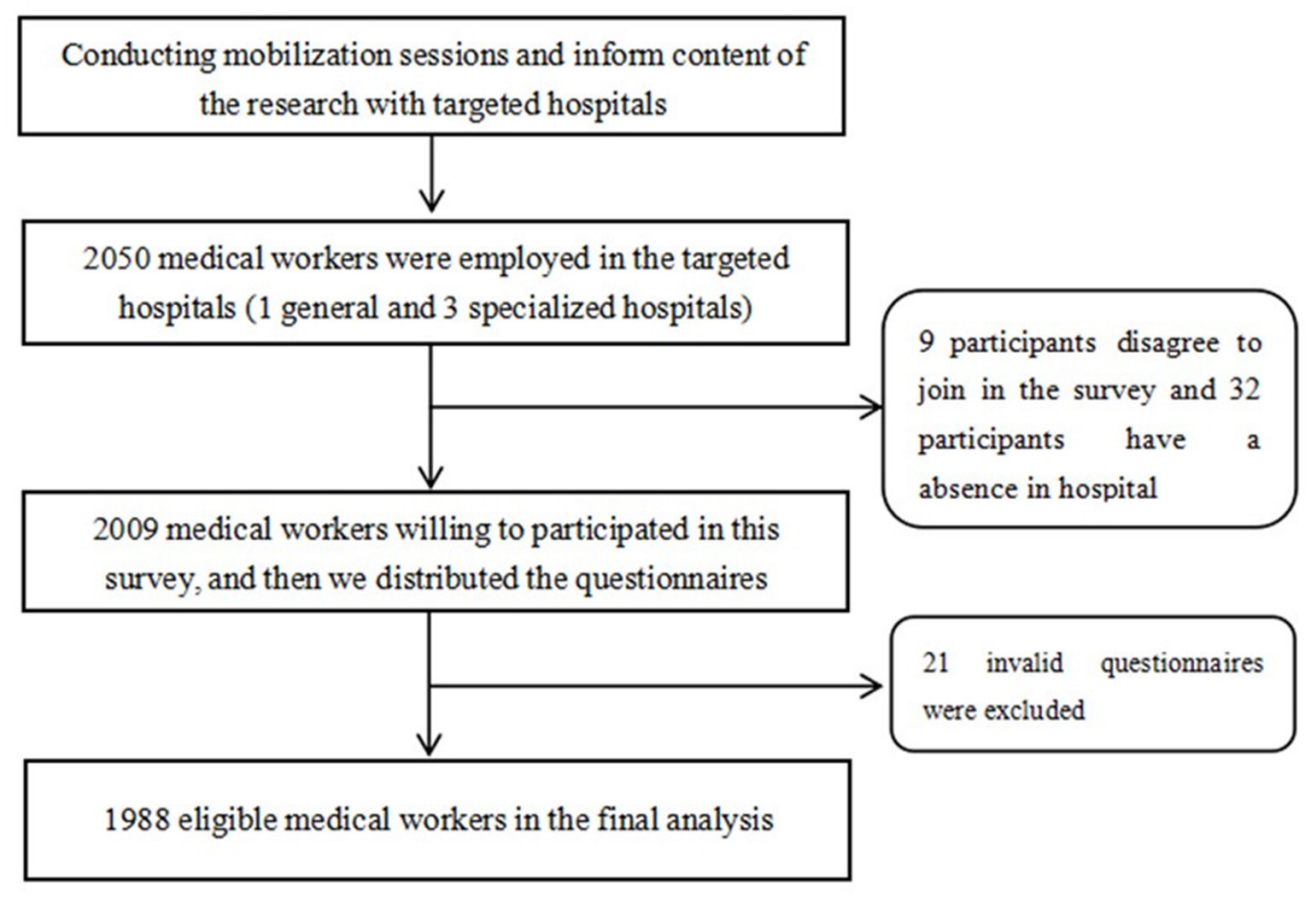
Study flowchart.

The inclusion criteria of this study were as follows: (1) individuals who had no family history of and were not taking medications for a mental disorder; (2) individuals who were regular employees of the targeted hospitals; and (3) individuals who were willing to participate in this survey. The exclusion criteria were as follows: (1) participants who were absent during the survey and (2) participants whose questionnaires were ineligible or were <80% completed. After written informed consent was obtained to conduct this study, a total of 2050 questionnaires were distributed. Nine participants did not agree to participate in the survey, thirty-two participants were absent from the hospital during the survey, and twenty-one participants returned incomplete (<80%) questionnaires. Finally, 1988 questionnaires were considered valid, corresponding to a response rate of 97.0% (1988/2050). The study flowchart is presented in Figure 1.

### Measurements

Researchers conducted a cross-sectional survey of medical workers at a general hospital and three specialty hospitals to evaluate the medical workers' WOF level, occupational stress and depression symptoms. The collection of basic information by self-administered basic information questionnaire was divided into demographic and occupational characteristics for each participant. The demographic characteristics included sex (male or female), age, marital status (single, cohabiting married, divorced and living alone, widowed and living alone), education (diploma or less, bachelor's degree or higher), monthly income (<3,000 CNY, 3,000–3,999 CNY, 4,000–4,999 CNY, 5,000–5,999 CNY, ≥6,000 CNY). The occupational characteristics included work experience, department, professional title, night shift per week, and work hours per week. The following survey instruments were used to collect data:

#### Measurement of Occupational Stress and Depression Symptoms

Depression, Anxiety, and Stress Scale (DASS-21), which was developed by Lovibond et al. (37) and has been proven to be a valid measure to assess stress in the Chinese population (38), which was used to measurement of occupational stress and depression symptoms. The DASS-21 is composed of 21 items, which are rated using a four-point Likert scale to assess the level of depression, anxiety, and stress. Each item was scored from 0 (it does not apply to me at all in the last week) to 3 (it applies to me perfectly in the last week). There are three sub-scales, included stress (seven items: 1, 6, 8, 11, 12, 14, and 18), depression (seven items: 3, 5, 10, 13, 16, 17, and 21), anxiety (seven items: 2, 4, 7, 9, 15, 19, and 20), respectively. In this study, we mainly used the stress sub-scale and depression sub-scale. DASS-21 scores were multiplied by two to calculate the final subscale scores, yielding a maximum of 42 points. Participants with a total score of 15 were rated as having occupational stress, the score of 10 was rated as having depression symptoms (37). We emphasized in the questionnaire that each response in the stress was related to work factors in hospitals.

#### Measurement of the Worker-Occupation Fit

WOF is an emerging and important factor for occupational health, which is still neglected in occupational stress research. The Worker-Occupation Fit Inventory (WOFI) is based on the questionnaire by Cable et al. (39), with some items and descriptions modified according to the Chinese culture and thinking to facilitate understanding by Chinese participants. The WOFI consists of three sections totalling nine items: (1) Characteristic Fit, for example, “Do you think your job style fits your job?”; (2) Need-Supply Fit, for example, “Do you think the job provides what you expect?”; and (3) Demand-Ability Fit, for example, “Do you think that your educational background meets the job demands?”. Each item is rated on a 5-point Likert scale (extremely unfit/fit, range 1–5). The higher the score is, the better the worker-occupation fit. Items used in this section of the survey are described in Supplementary Table S1. In this study, Cronbach's alpha coefficient of the total scale was 0.888. Cronbach's alpha coefficients of CF, NSF, and DAF were 0.790, 0.848, and 0.895, respectively. The result of test-retest reliability is 0.897.

### Data Analysis

**Figure 2:**
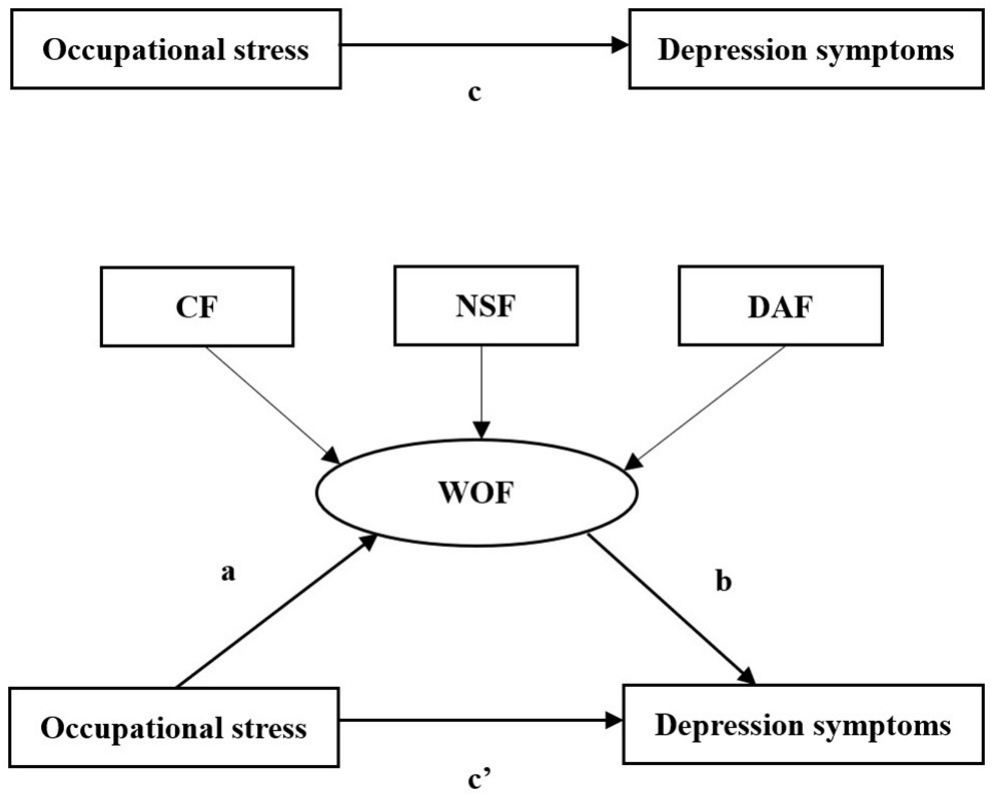
The conceptional model on the mediating role of WOF on the association between occupational stress and depression symptoms. WOF, worker occupation fit.

The demographic and occupational characteristics of the medical workers are described by the frequency and percentage. The categorical variables are expressed as frequencies (%) and were compared by Chi-square tests among all participants. Descriptive statistics for the level of occupational stress, depression symptoms and WOF were showed with mean, standard deviation (*SD*). The correlation between occupational stress, depression symptoms and WOF was used by Pearson's correlation analysis. Before regression analyses, all the continuous variables in the models were centralized. The conceptual framework of the study is shown in Figure 2, the effect of occupational stress on depression symptoms would have two path hypotheses. Hypothesis 1, the aim was to verify the direct effect of occupational stress on depression symptoms (the c-path) after adjusting covariates; the hypothesis 2, the aim was to verify the mediating role of WOF between occupational stress and depression symptoms. We performed a hierarchical linear analysis for each of the WOF and the WOF types to test the mediating effects on depression symptoms. At the first step, the variables have a significant association with depression symptoms by univariate analysis that those of them as the control variable in block 1. At the second step, occupational stress was added as an independent variable and a mediating variable in block 2. At the third step, WOF and WOF types (CF, NSF and DAF) entered into block 3, respectively. Besides, the bootstrap estimate was based on 2,000 bootstrap samples. A bias-corrected and accelerated 95% confidence interval (BCa 95% CI) was calculated for each a×b product, and a BCa 95% CI excluding 0 significantly manifested mediation. All statistical analyses were carried out using *R* Statistics (version 3.5.0), with a two-tailed probability value of <0.05 considered to be statistically significant.

## Results

### The Participants Characteristic

Demographic and occupational characteristics of the positive cases of depression symptoms in categorical variables are shown in Table 1. In this study, the average age of participants was 32.7 (*SD* = 7.8). There were a total of 1988 medical workers, 43.5% (*n* = 864) of the medical workers experienced depression symptoms. The mean score of WOF was 31.6 ± 7.1, CF, NSF and DAF were 11.3 ± 2.5, 10.1 ± 2.7, 12.9 ± 2.2, respectively.

**Table 1:** Demographic and occupational characteristics of participants (*n* = 1988) and comparisons on the positive case of depression symptoms.

**Variable**	***n***	**Positive**		***P***
		**cases**	**%**	
Occupation				0.097
Physician	525	212	40.4	
Nurse	1,463	652	44.6	
Sex				0.006
Male	214	112	52.3	
Female	1,774	752	42.4	
Marital status				0.216
Single	511	207	40.5	
Cohabit	19	115	7.9	
Married	1,423	631	44.3	
Divorce and live alone	31	12	38.7	
Widowhood and live alone	4	3	75.0	
Education				<0.001
Bachelor	469	172	36.7	
Master	1,452	653	45.0	
Doctor	67	39	58.2	
Income				0.005
<3,000 CNY	334	119	35.6	
3,000–3,999 CNY	686	290	42.3	
4,000–4,999 CNY	450	204	45.3	
5,000–5,999 CNY	315	150	47.6	
≥6,000 CNY	203	101	49.8	
Work experience				<0.001
<1 year	150	42	28.0	
1–3 years	297	117	39.4	
4–10 years	757	355	46.9	
11–19 years	512	242	47.3	
≥20 years	272	108	39.7	
Department of				0.003
Internal Medicine	355	171	48.2	
Surgery	325	148	45.5	
Obstetrics and Gynecology	237	91	38.4	
Pediatric Surgery	83	44	53.0	
Psychiatry	248	108	43.6	
Infectious Disease	111	34	30.6	
Emergency	140	72	51.4	
ICU	136	62	45.6	
Outpatient Clinic	79	29	36.7	
Others	274	105	38.3	
Title				0.019
Primary	1,125	458	40.7	
Intermediate	756	356	47.1	
Senior	107	50	46.7	
Night shift per week				<0.001
No	691	256	37.1	
≥2 times	194	77	39.7	
≥4 times	175	82	46.9	
≥6 times	928	449	48.4	
Work hour per week				<0.001
<40 hours	534	196	36.7	
40–49 hours	940	396	42.1	
50–59 hours	300	142	47.3	
≥60 hours	214	130	60.8	

### Correlation of Occupational Stress, Depression Symptoms and WOF

The results of the Pearson's correlation analyses are showed in Table 2. Occupational stress, depression symptoms, and WOF were significant correlated. The occupational stress was negatively related to WOF (*r* = −0.395, *P* < 0.001), CF (*r* = −0.529, *P* < 0.001), NSF (*r* = −0.500, *P* < 0.001), and DAF (*r* = −0.345, *P* < 0.001). The occupational stress and depression symptoms have a positive relationship (*r* = 0.798, *P* < 0.001). Based on these results, the paths were drawn to examine the mediating effect (Figure 2). And there was a correlation between occupational stress and WOF (CF, NSF, and DAF) (path a). The WOF was correlated with depression symptoms (path b), whereas occupational stress was correlated with depression symptoms (path c).

**Table 2:** The correlations of depression symptoms, occupational stress, WOF and WOF types.

**Variable**	**1**	**2**	**3**	**4**	**5**	**6**
1. WOF	1					
2. CF	0.593**	1				
3. NSF	0.580**	0.690**	1			
4. DAF	0.496**	0.520**	0.373**	1		
5. Depression symptoms	−0.395**	−0.529**	−0.500**	-0.345**	1	
6. Occupational stress	−0.259**	−0.431**	−0.428**	-0.258**	0.798**	1

*WOF, worker-occupation fit; CF, Characteristic fit; NSF, need supply fit; DAF, demand ability fit.*

** *Correlation is significant at the 0.001 level (two-tailed)*.

### Mediation Effect Test

**Figure 3:**
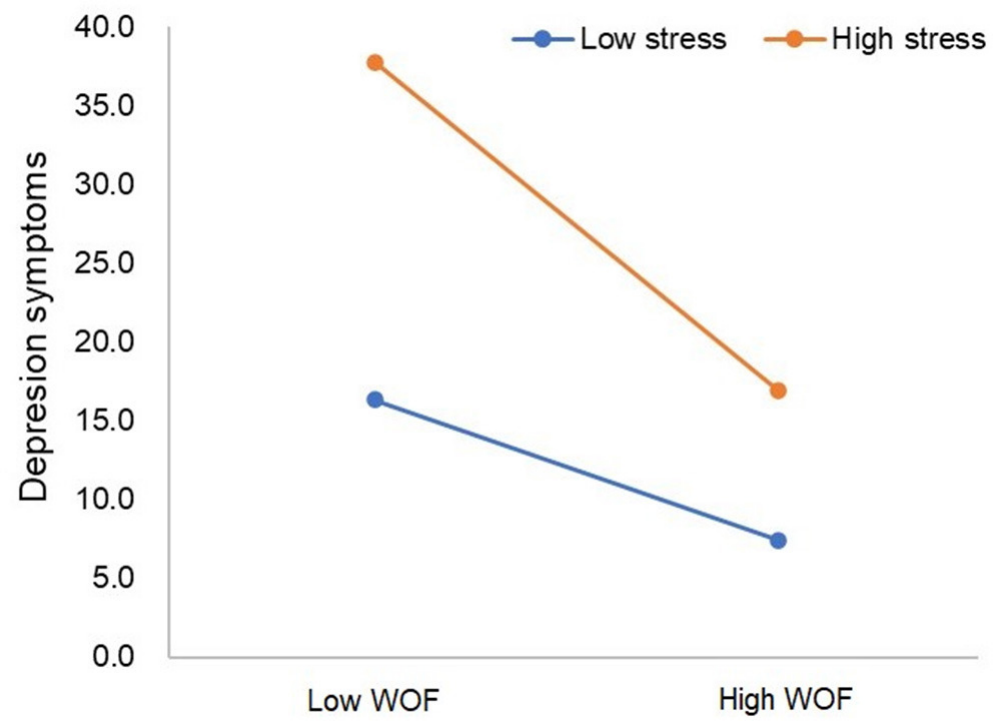
WOF moderates the effect of occupational stress on depression symptoms based on the Block 3-Model 1.

The result of the hierarchical regression of depression symptoms is shown in Table 3. Based on the Table 3's block 3-model 1, Figure 3 shows the WOF mediates the effect of occupational stress on depression symptoms. Each step of the independent variables made a significant contribution to variance in depression symptoms. In step 1, the occupational categories, sex, age, and work experience contributed to 4.6% of the variance in depression symptoms. The dimensions of occupational stress accounted for 64.4% of the variance in depression symptoms in step 2. The results supported that occupational stress was positively related to depression symptoms (β = 0.805, *P* < 0.001). In block 3 and model 1, WOF was negatively related to depression symptoms (β = −0.290, *P* < 0.001, R^2^ = 0.684). In block 3 and model 2, when the WOF types into the model, the results shown that WOF types were negatively related to depression symptoms, the regression coefficient were −0.114, −0.114, −0.048 and −0.034, respectively (R^2^ = 0.692, *P* < 0.001).

**Table 3:** Hierarchical multiple regression analysis of the association of occupational stress and WOF with depression symptoms.

**Variable**	**Block 1**		**Block 2**		**Block 3**			
					**Model 1**		**Model 2**	
	**β**	**VIF**	**β**	**VIF**	**β**	**VIF**	**β**	**VIF**
Occupational categories	0.088	1.22	−0.062	1.26	0.136	2.23	0.021	4.28
Sex (female)	−0.036	1.08	−0.008	1.08	−0.006	1.08	−0.007	1.08
Age (year)	−0.080	4.58	0.042	4.61	0.039	4.61	0.044	4.63
Work experience (year)	0.157	4.53	−0.009	4.57	−0.019	4.58	−0.022	4.58
Occupational stress			0.805	1.08	0.702	1.35	0.688	1.39
WOF					−0.290	2.10	−0.114	7.03
CF							−0.114	2.86
NSF							−0.048	2.65
DAF							−0.034	1.82
*Wald χ^2^*	116.94		2,864.27		3,627.30		3,944.34	
R^2^	0.046		0.644		0.684		0.692	
ΔR^2^	0.041		0.642		0.683		0.690	
MSE	7.290		4.451		4.193		4.146	

*WOF, worker occupation fit; CF, Characteristic fit; NSF, need supply fit; DAF, demand ability fit*.

**Figure 4:**
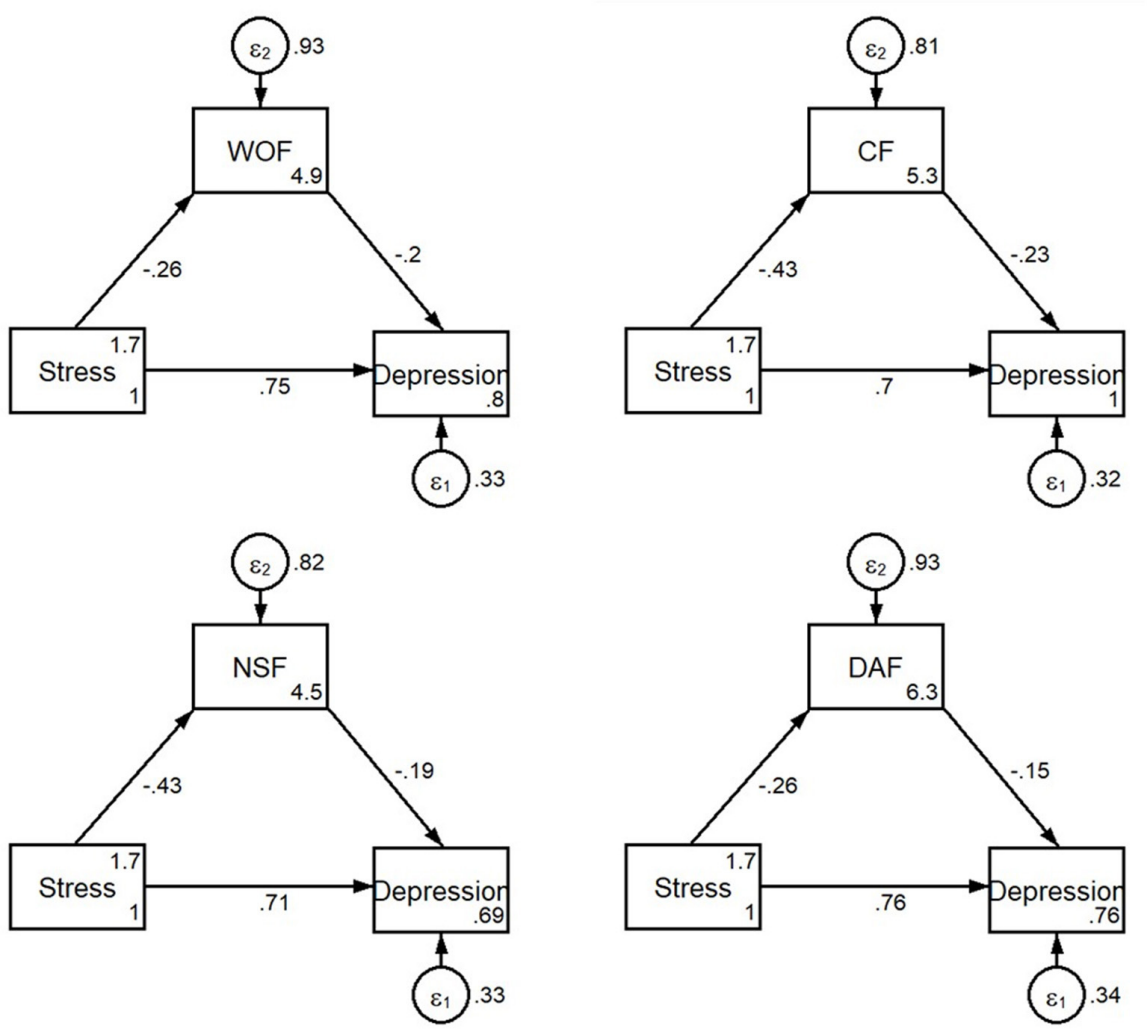
The structural equation model on the relationship between occupational stress, depressive symptoms, WOF and WOF types among 1988 medical workers. Stress, occupational stress; Depression, depression symptoms. WOF, worker occupation fit; CF, Characteristic fit; NSF, need supply fit; DAF, demand ability fit.

The results for the direct and indirect effects of occupational stress on depression symptoms with WOF and WOF types as mediators are presented in Table 4. The standardized estimates of the path coefficients for each variable are shown in Figure 4. SEM revealed significant regression or correlation paths, and all beta path coefficients were statistically significant (*P* < 0.001). SEM revealed significant regression or correlation paths, and all beta path coefficients were statistically significant (*P* < 0.01). The WOF to be a mediator, occupational stress had a direct effect and an indirect effect on depression symptoms with a path coefficient of 0.677 (*P* < 0.01) and 0.047 (*P* < 0.01), respectively. The CF to be a mediator, occupational stress had a direct effect and an indirect effect on depression symptoms with a path coefficient of 0.636 (*P* < 0.01) and 0.089 (*P* < 0.01), respectively. The NSF to be a mediator, occupational stress had a direct effect and an indirect effect on depression symptoms with a path coefficient of 0.650 (*P* < 0.01) and 0.075 (*P* < 0.01), respectively. In addition, the DAF to be a mediator, occupational stress had a direct effect and an indirect effect on depression symptoms with a path coefficient of 0.690 (*P* < 0.01) and 0.035 (*P* < 0.01), respectively. We also calculated that the total effect of occupational stress on depression symptoms was 93.4% and the indirect effect accounted for 6.5% of the total effect. As shown in Table 4, the bootstrap estimate presented in our study was based upon 2,000 bootstrap samples. For each independent variable, the bias-corrected 95% CI all excluded 0, which indicated that the mediating role of WOF was statistically significant.

**Table 4:** Mediating role of WOF or WOF types on the associations between occupational stress and depression symptoms.

**Mediators**	**Model pathways**	**Effect types**	**β(std.)**	**Bootstrap**	
				**Bias-corrected 95%**	
				**Lower bounds**	**Upper bounds**
WOF					
	Occupational stress → Depression symptoms	Total effects	0.725	0.692	0.753
		Indirect effects	0.047	0.039	0.056
		Direct effects	0.677	0.648	0.707
CF	Occupational stress → Depression symptoms				
		Total effects	0.725	0.695	0.754
		Indirect effects	0.089	0.074	0.104
		Direct effects	0.636	0.603	0.668
NSF	Occupational stress → Depression symptoms				
		Total effects	0.725	0.696	0.754
		Indirect effects	0.075	0.062	0.089
		Direct effects	0.650	0.617	0.682
DAF	Occupational stress → Depression symptoms				
		Total effects	0.725	0.695	0.755
		Indirect effects	0.035	0.026	0.044
		Direct effects	0.690	0.658	0.722

*WOF, worker occupation fit; CF, Characteristic fit; NSF, need supply fit; DAF, demand ability fit.*

*The mediating effect of WOF, CF, NSF, and DAF accounted for 6.5, 12.3, 10.3 and 4.8%, respectively*.

## Discussion

Improving WOF level was considered as a new approach to prevention of depression symptoms in workplace. This study aims to examine the relationship between occupational stress and depression symptoms and whether the WOF is a mediator into this relationship. In this study, there are 43.5% (864/1,988) medical workers with a positive rate of depression symptoms, and the average score of WOF was 31.6 ± 7.1. The WOF to be a mediator, occupational stress had a direct effect and an indirect effect on depression symptoms with a path coefficient of 0.677 and 0.047. The mediating effect of WOF, CF, NSF, and DAF accounted for 6.5, 12.3, 10.3 and 4.8%, respectively.

According to the World Health Organization (WHO), nearly 300 million people of different ages suffer from depression worldwide. In China, medical workers have reported high rates of depression (50%), anxiety (45%), and insomnia (34%) (40). The prevalence of depression increased by more than 18% from 2005 to 2015 (41). Among emergency medical service workers at the New York City Fire Service (FDNY), the prevalence of depression symptoms was 16.7% after 12 years of occupational exposure (42). Therefore, depression and anxiety have become a global consensus for medical workers. Medical workers are one of the highest risk groups for developing depression (43). We try to find effective ways to reduce the risk of depression among medical workers. In this sense, when medical workers experience depression symptoms in an occupational environment, improve the WOF level is the regulation process against depression, and can be an effective factor in maintaining mental health. In our study, the participants were selected from one general hospital and three specialized hospitals located in Henan province, with a detection rate of depression symptoms were 43.5%. Compared to that of medical students (44), firefighters (45), economy staff (46), medical workers have a higher detection rate of depression symptoms.

Occupational stress has been identified as a risk factor for poor mental health and depression symptoms. To develop effective interventions, the roles of positive predictors and mediators should be explored to clarify the mechanism behind the association between occupational stress and depression symptoms. The most important goal of this study was to examine the relationships between depression symptoms, occupational stress, WOF. Our results show that the WOF types (CF, NSF, and DAF) were negatively correlated with depression symptoms, and the occupational stress and depression symptoms was a positive relationship with 1988 medical workers. Practical strategies for improving medical workers' WOF would help them better cope with various worker-related stressors to reduce depression symptoms. In our study, the structural model analysis indicated that occupational stress had both direct and indirect effects on depression symptoms with WOF considering as mediators. In other words, occupational stress indirectly affected depression symptoms by decreasing WOF, it was implied that WOF could mediate the association between occupational stress and depression symptoms. These results suggested that the WOF level are crucial for the mental and physical health of medical workers. In addition, results from block 3 (model 1 and model 2) by hierarchical regression, based on the absolute value of β, CF, NSF and DAF accounted for the observed variance in WOF. It is noteworthy that CF is more strongly associated with WOF than with NSF, DAF. The results suggest that hospital administrators assign work tasks based on the personal characteristics (i.e., personality or work style) of medical staff, which can reduce the risk of depression symptoms. In a systematic review, the study indicated a high prevalence of depression among medical staff and the consistency between the estimated prevalence (27.2% students and 28.8% residents) showed that depression coping was an important issue in all levels of medical training (47). In this sense, medical workers with depression should be given an early warning. The hospital administrators can reduce medical workers' depression symptoms by taking comprehensive measures to improve the WOF such as establishing training sessions to improve the ability of medical workers to meet the hospital demands, promoting the balance between demands and rewards (48). It can also improve WOF level by creating a better fit between workers' characters and hospitals' climate, workers' ability and hospitals' demands, workers' needs and hospitals' supply.

Previous studies of organizational behavior and organizational management showed that high-level fit of worker and occupational environment can improve work attitude (49), innovation behaviors (50), and organizational commitment (51), and turnover rate (52). It has a positive effect on improving the optimization of the organizational outcome. The results of our study suggested WOF, occupational stress and depression symptoms have a significant correlation, and WOF can explain 6.5% of the effect of occupational stress on depression symptoms. A better WOF level can promote medical workers' characters and habits more suitable for their job style in hospitals, and workers' medical service skills can skillfully complete the tasks assigned by their managers, which would reduction of the medical workers' occupational stress, thereby reducing the depression symptoms. The current study is one of the few to investigate WOF among Chinese medical workers, and to examine the correlation between occupational stress and depression symptoms, and it is the first study to explore the mediating role of WOF in these relationships. In China, basic salary levels are generally low, hospital managers tend to use bonuses to incentivize their employees, which is usually linked to work outputs rather than work experience. The low income of health workers has been a serious challenge in many developing countries (53). For medical workers, it is important to note that strategies to enhance individuals' WOF seems to be valuable to improve depression symptoms and promote the mental health of Chinese medical workers.

To our knowledge, this is one of the few studies to explore the effects of occupational stress, WOF on depression symptoms, to further examined the mediating roles of WOF among Chinese medical workers. It is provided with a new perspective for exploring the relationship between occupational stress and depression symptoms. However, there are two limitations: firstly, a cross-sectional study was conducted. The causal relationship between occupational stress, depression symptoms and WOF cannot be identified. Secondly, all of the medical workers were from one general hospital and three specialized hospitals in Henan Province, and the sample was not randomized, which suggested the generalization of results to other areas of China would be limited.

## Conclusion

In conclusion, WOF was found to be negatively associated with depression symptoms and partially mediated the relationship between occupational stress and depression symptoms. These results could help to further explore the relationship between occupational stress, depression symptoms and WOF, as well as a new perspective for the intervention of depression symptoms. Hospital managers can help medical workers coping with various worker-related stressors to reduce depression symptoms.

## Data Availability Statement

The raw data supporting the conclusions of this article will be made available by the authors, without undue reservation.

## Ethics Statement

The studies involving human participants were reviewed and approved by the Ethics Committees of West China School of Public Health and West China Fourth Hospital. The patients/participants provided their written informed consent to participate in this study.

## Author Contributions

RS: study design, data analysis, data interpretation, and finished the manuscript. RS, ZH, LL, and HW: data acquisition. RS, KL, and YL: data analysis. YL: supervision of study. All authors have read and agreed to the published version of the manuscript. All authors contributed to the article and approved the submitted version.

## Funding

This study was funded by National Natural Science Foundation of China (Grant Number: 82073521) and the Natural Science Foundation of Chengdu Medical Collage (Grant Number: CYZYB21-32). The funders have no role in the analysis, or interpretation of the data, writing of the article, or the decision to submit the paper for publication.

## Conflict of Interest

The authors declare that the research was conducted in the absence of any commercial or financial relationships that could be construed as a potential conflict of interest.

## Publisher's Note

All claims expressed in this article are solely those of the authors and do not necessarily represent those of their affiliated organizations, or those of the publisher, the editors and the reviewers. Any product that may be evaluated in this article, or claim that may be made by its manufacturer, is not guaranteed or endorsed by the publisher.
