# Supplementary material for: The Mediating Role of Worker-Occupation Fit in the Relationship Between Occupational Stress and Depression Symptoms in 1988 Medical Workers: A Cross-Sectional Study
Source: Front Public Health. 2022 May 17;10:843845. doi: 10.3389/fpubh.2022.843845 (PMC9152027; doi:10.3389/fpubh.2022.843845)
Supplement: Supplementary file 1 [file Table_1.DOCX]

**Supplementary material-Table S1. The items of The Worker Occupation Fit Inventory (WOFI)**

| **Dimensions** | **Items** | **Rating Scale** |
| --- | --- | --- |
| Characteristic fit (CF) | 1. The personality/ habit fit in the job | Five-point scale ranging from (1) extremely misfit to (5) extremely fit |
|  | 2. Personal values fit in the work atmosphere |  |
|  | 3. Physical capability fit in the job demand |  |
| Need supply fit (NSF) | 4. The job provide what you material and spiritual need |  |
|  | 5. The job provide comfortable working environment |  |
|  | 6. The job provide the sense of satisfaction |  |
| Demand ability fit (DAF) | 7. Educational Background match with job’s demand |  |
|  | 8. Theoretical skills match with job’s demand |  |
|  | 9. Technical skills match with job’s demand |  |
